# Supplementary material for: A three-order-parameter bistable magnetoelectric multiferroic metal
Source: Nat Commun. 2020 Oct 1;11:4922. doi: 10.1038/s41467-020-18664-6 (PMC7530708; doi:10.1038/s41467-020-18664-6)
Supplement: Supplementary file 1 — Supplementary Information [file 41467_2020_18664_MOESM1_ESM.pdf]

SUPPLEMENTARY INFORMATION
A three-order-parameter bistable magnetoelectric multiferroic metal

Andrea Urru,[1,2] Francesco Ricci,[3] Alessio Filippetti,[1] Jorge Íñiguez,[4,5] and Vincenzo Fiorentini[1]

[1]*Dipartimento di Fisica, Università di Cagliari, Cittadella Universitaria, Monserrato, I-09042 Cagliari, Italy*
[2]*Scuola Superiore Internazionale di Studi Avanzati, Via Bonomea 265, I-34136 Trieste, Italy*
[3]*Institute of Condensed Matter and Nanosciences (IMCN), Université Catholique de Louvain, Chemin des Étoiles 8, B-1348 Louvain-la-Neuve, Belgium*
[4]*Materials Research and Technology Department, Luxembourg Institute of Science and Technology, 5 avenue des Hauts-Fourneaux, L-4362 Esch/Alzette, Luxembourg*
[5]*Department of Physics and Materials Science, University of Luxembourg, 41 Rue du Brill, L-4408 Belvaux, Luxembourg*

(Dated: September 3, 2020)

SUPPLEMENTARY METHODS

Ab initio Density Functional Theory calculations have been performed within the Local Density (LDA) and Generalized Gradient (GGA) Approximations for exchange and correlation, as implemented in `VASP` and `Quantum ESPRESSO` (QE), and supported by variational pseudo-self-interaction-corrected (`VPSIC`) GGA calculations.

We identified the instabilities in the high-symmetry ($Pmnn$) phase by diagonalizing the $\mathbf{q}$=0 dynamical matrix, computed both with finite differences (VASP) and Density Functional Perturbation Theory (QE) methods within GGA. Structural relaxations for the $Pmnn$, $Pmn2_1$, and $Pm2_1n$ phases have been carried out with both VASP and QE in both GGA and LDA. Collinear magnetism calculations used to estimate the exchange parameter have been performed within GGA.

Non-collinear magnetism and anisotropy energies for the lattice directions have been studied both within GGA (VASP) and LDA (QE), with fully consistent results. For computational expediency we used LDA to map out the anisotropy energy in generic directions.

Convergence tests have been performed with both codes separately. The ion cores are described by projector augmented waves (VASP) or ultrasoft (VPSIC, QE) pseudopotentials. In non-collinear calculations in QE, spin-orbit coupling has been included by using fully relativistic (FR) pseudopotentials. The pseudo-wavefunctions have been expanded in a plane waves basis set with a kinetic energy cut-off of 500 eV (VASP), 476 eV (VPSIC), and 90 Ry (QE).

The Brillouin Zone integrations have been done using a $6 \times 2 \times 4$ Monkhorst-Pack mesh for self-consistent field and structural optimization calculations, a $12 \times 4 \times 8$ mesh to compute densities of states, and a 8×4 set of 11-$\boldsymbol{k}$-point strings to compute the electronic polarization. The Fermi surface has been smeared by the gaussian method, with a smearing parameter $\sigma = 0.1$ eV.

SUPPLEMENTARY DISCUSSION

Structural relaxations results for the $Pmnn$, $Pmn2_1$, and $Pm2_1n$ phases (carried out with both VASP and QE in GGA and LDA) are summarized in Supplementary Table I. The GGA results in VASP and QE are very close; as expected LDA lattice constants are smaller than GGA ones, but this hardly affects lattice displacements, bands, and magnetism.

$Pmnn$ phase			
Exchange-correlation functional	a (Å)	b (Å)	c (Å)
GGA (VASP)	3.93	30.65	5.40
GGA (QE)	3.93	30.65	5.40
$Pmn2_1$ phase			
GGA (VASP)	3.92	30.71	5.42
GGA (QE)	3.92	30.69	5.41
FR-LDA (QE)	3.85	29.87	5.26

Supplementary Table I. GGA and fully-relativistic LDA lattice constants of the $Pmnn$ and $Pmn2_1$ phases.

To monitor the effects of the Hubbard U parameter on Mn ions, GGA+U calculations have been carried out as well, with U ranging from 1 to 5 eV (the typical literature value is 3 eV); this results, as mentioned in the main text, in an absolute-value increase of the J parameter, as shown in Supplementary Figure 1. Improved electronic structure beyond semilocal functionals, besides all the polarization properties, have been computed with VPSIC.

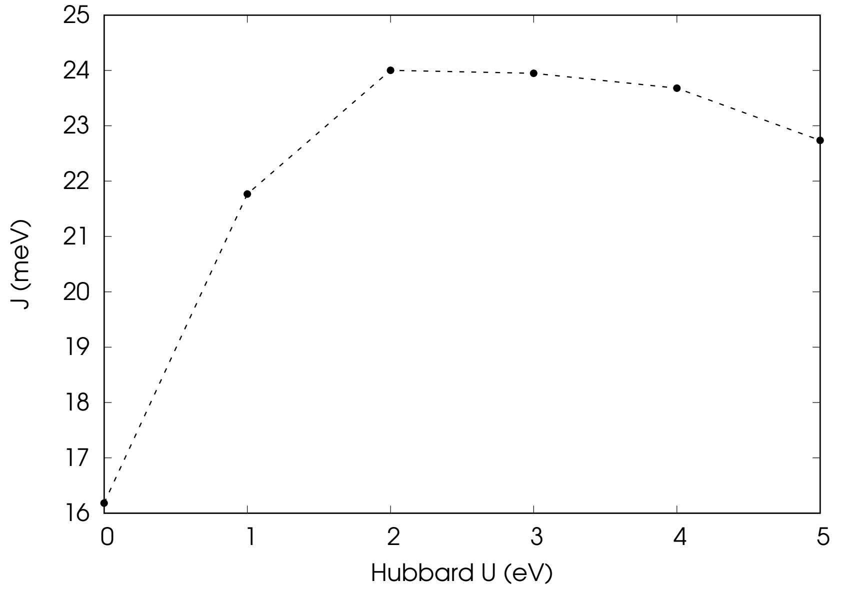

Supplementary Fig. 1. Exchange parameter J for different values of the Hubbard U parameter.

We now briefly discuss a minimal Landau free energy model with trilinear coupling. As described in the main text, BiMO has three order parameters. Due to the symmetry described by the magnetic point groups of the two low-symmetry phases, namely $m2'm'$ and $m'm2'$ for the B and C states, the toroidization $\mathbf{T}$ must be orthogonal to both the polarization $\mathbf{P}$ and the magnetization $\mathbf{M}$, and hence we can write $\mathbf{T}\propto\mathbf{P}\times\mathbf{M}$. Such a relationship arises if the free energy of the multiferroic phase contains a trilinear coupling term $\alpha\,\mathbf{T}\cdot[\mathbf{P}\times\mathbf{M}]$. In BiMO, given the results discussed below, we can assume that $\mathbf{P}$ and $\mathbf{M}$ are the primary order parameters, while $\mathbf{T}$ is a secondary order parameter that stems from the simultaneous breaking of time-reversal and inversion symmetry in the low-symmetry phase. As a consequence, $\mathbf{T}$ can be considered as a slave variable in a Landau expansion of the free energy:

$$F = F_0 + a_1\mathrm{M}^2 + a_2\mathrm{P}^2 + a_3\mathrm{T}^2 + c_{11}\mathrm{M}^4 + c_{22}\mathrm{P}^4 + c_{12}\mathrm{M}^2\mathrm{P}^2 + \alpha\mathrm{M\ P\ T}. \tag{1}$$

While a detailed discussion is outside the scope of this work, the trilinear coupling has a few immediate consequences. The stationarity condition $\boldsymbol{\nabla}F = 0$ leads to

$$\mathrm{M} = \pm\mathrm{M}_0 \tag{2}$$
$$\mathrm{P} = \pm\mathrm{P}_0 \tag{3}$$
$$\mathrm{T} = -\frac{\alpha}{2a_3}\mathrm{M\ P} \tag{4}$$

with $\pm\mathrm{M}_0$ and $\pm\mathrm{P}_0$ the roots of the equations

$$0 = \left.\frac{\partial F}{\partial \mathrm{M}}\right|_{P=P_0}, \qquad 0 = \left.\frac{\partial F}{\partial \mathrm{P}}\right|_{M=M_0}.$$

While P and M do not depend on T and can be non-zero, T is completely determined by P and M and by the sign of α through Eq. (4). As a result, the stationarity condition provides four solutions, as summarized in Table II.

From Eq. (4), it follows that $\mathrm{T} = 0$ if $\alpha = 0$. On the other hand, if $\alpha \neq 0$, toroidal order arises, and in particular the trilinear coupling has a stabilizing effect on the group of four states in the right column of Table II corresponding to negative α. The four states are degenerate in energy, as determined by Eq. (1).

In our case, as mentioned in the main text, we verified that in BiMO $\mathrm{T}\neq 0$ and that, further, the four states in Table II, right column, do exist and are indeed degenerate, within 2 μeV per Mn atom in the worst case (P-reversal has

$\alpha > 0$				$\alpha < 0$		
M	P	T		M	P	T
+	+	−		+	+	+
+	−	+		+	−	−
−	+	+		−	+	−
−	−	−		−	−	+

Supplementary Table II. Sign of the solutions of $\nabla F = 0$ for $\alpha > 0$ and $\alpha < 0$.

been obtained by reverting the atomic displacements that lead from the high-symmetry to the low-symmetry phase, and by further relaxing the structure.). These results are evidence that a stabilizing trilinear coupling $\alpha < 0$ exists between the order parameters of BiMO.

This conclusion is also supported by further numerical investigations on the origin of **T** from the Mn magnetic moments. Since **T**=0 in the centrosymmetric high-symmetry phase, the toroidization of the low-symmetry phase is to leading order

$$\mathbf{T} = \frac{1}{2\Omega} \sum_{i \in \mathrm{Mn}} \left(\Delta \boldsymbol{r_i} \times \boldsymbol{m_i} + \boldsymbol{r_i} \times \Delta \boldsymbol{m_i} \right), \tag{5}$$

where Ω is the unit cell volume and $\Delta \boldsymbol{r_i}$ and $\Delta \boldsymbol{m_i}$ are, respectively, the atomic displacements and the magnetic moment changes occurring as the $Pmnn$ phase distorts into $Pmn2_1$ or $Pm2_1n$. The $\Delta \boldsymbol{m_i}$ comprise slight changes of the magnitude of $\boldsymbol{m_i}$ and, predominantly, any canting out of the easy axis consistent with the magnetic point symmetry. We find that in BiMO the canting of the magnetic moments is the main source of **T**, with the second term of Eq.(5) accounting for around 90% of the toroidization. As a consequence, in BiMO the sign of **T** can be mainly controlled by the canting vectors $\Delta \boldsymbol{m_i}$: specifically, the sign of the canting vector $\Delta \boldsymbol{m_i}$ (of given direction and modulus) determines the sign of **T**. The cantings do not affect **M**, hence in principle it would be possible to revert **T**, with fixed **P** and **M**, by reverting the sign of all the vectors $\Delta \boldsymbol{m_i}$. However, such "counter-canting" would be suppressed by the trilinear coupling term, which would cant the moments back to their stable configuration. We verified that indeed the configuration (−P, +M, +T), obtained from (−P, +M, −T) by inverting all the $\Delta \boldsymbol{m_i}$, is unstable and the system reverts to the state (−P, +M, −T).

In summary, the sign of the order parameters **P** and **M** can be controlled externally, while **T** follows mostly the internal rearrangement of the magnetic moments, and to a lesser extent the polar displacements. It is then natural to describe **T** as a secondary order parameter determined (in the model described by Eq.(1)) by P, M, and α.
